# The Possible Effects on Socio-Economic Inequalities of Introducing HPV Testing as Primary Test in Cervical Cancer Screening Programs

**DOI:** 10.3389/fonc.2014.00020

**Published:** 2014-02-10

**Authors:** Paolo Giorgi Rossi, Flavia Baldacchini, Guglielmo Ronco

**Affiliations:** ^1^Servizio Interaziendale di Epidemiologia, Azienda Unità Sanitaria Locale di Reggio Emilia, Reggio Emilia, Italy; ^2^Unit of Cancer Epidemiology II, Center for Cancer Epidemiology and Prevention (CPO), Turin, Italy

**Keywords:** human papillomavirus, mass screening, social inequalities, participation, coverage, compliance

## Abstract

**Background:** Screening with HPV is more effective than Pap test in preventing cervical cancer. HPV as primary test will imply longer intervals and a triage test for HPV positive women. It will also permit the development of self-sampling devices. These innovations may affect population coverage, participation, and compliance to protocols, and likely in a different way for less educated, poorer, and disadvantaged women.

**Aim:** To describe the impact on inequalities, actual or presumed, of the introduction of HPV-based screening.

**Methods:** The putative HPV-based screening algorithm has been analyzed to identify critical points for inequalities. A systematic review of the literature has been conducted searching PubMed on HPV screening coverage, participation, and compliance. Results were summarized in a narrative synthesis.

**Results:** Knowledge about HPV and cervical cancer was lower in women with low socio-economic status and in disadvantaged groups. A correct communication can reduce differences. Longer intervals will make it easier to achieve high-population coverage, but higher cost of the test in private providers could reduce the use of opportunistic screening by disadvantaged women. There are some evidences that inviting for HPV test instead of Pap increases participation, but there are no data on social differences. Self-sampling devices are effective in increasing participation and coverage. Some studies showed that the acceptability of self-sampling is higher in more educated women, but there is also an effect on hard-to-reach women. Communication of HPV positivity may increase anxiety and impact on sexual behaviors, the effect is stronger in low educated and disadvantaged women. Finally, many studies found indirect evidence that unvaccinated women are or will be more probably under-screened.

**Conclusion:** The introduction of HPV test may increase population coverage, but non-compliance to protocols and interaction with opportunistic screening can increase the existing inequalities.

## Background

Cervical cancer is still the fourth cancer worldwide in terms of incidence, although the burden of disease is not evenly distributed, with about 80% of cases occurring in low-income countries (1). In industrialized countries, instead, incidence and mortality have been dramatically reduced thanks to Pap test and screening programs (2, 3). In fact, the Pap test is able to identify cytological abnormalities exfoliated from pre-cancerous lesions, and progression to cervical cancer is prevented through outpatient treatment (2, 3).

Persistent infection with HPV oncogenic types has been demonstrated to be the necessary, but not sufficient, cause of cervical cancer (3). Knowledge of the natural history of the disease has led to the introduction of two new tools for cervical cancer prevention: vaccine and HPV test for screening (4).

Population-based randomized trials have shown that screening with HPV as primary test is more effective than Pap test in reducing cervical cancer incidence (5) and mortality (6).

The accuracy characteristics of the HPV DNA test are different from those of the Pap test. First and foremost, the former is more sensitive and less specific (7). Further, it has a higher prospective negative predictive value, i.e., the risk of having a CIN3 or cancer 5 years after an HPV-negative test is still lower than the risk 3 years after a negative Pap test (5, 8). Due to lower specificity a higher number of women will need further ascertainment. However, few of them will have a high-grade lesion and direct referral to colposcopy may thus be too intensive an approach (9–11). On the other hand, higher sensitivity and prospective negative predictive value will allow longer intervals (5, 8). HPV infection can persist for several years before its clearance or progression to a high-grade lesion requiring treatment; women with long-term persistent infections need to be followed up with reasonable protocols, adapted to their risk of developing cancer (12).

Therefore the shift from Pap test to HPV test as a primary screening test will dramatically change the screening program protocols and organization: the intervals will be longer, and a triage test for HPV positive women and more complex algorithms for the management of positive women will be needed. Furthermore, the introduction of HPV test will result in less intensive protocols and follow up. Finally, a molecular test makes it possible to develop self-sampling devices.

All these changes and technical innovations may affect screening participation and population coverage (11, 13), and most likely in a different way for less educated, poorer, and more disadvantaged women (14–16).

To date, only few countries have introduced the HPV test in the organized screening protocol and none has yet implemented such protocols at a national level; several countries have large-scale population-based pilot projects under way (17–21).

## Objective

To identify the possible effect of the introduction of HPV-based protocols in organized screening program on social inequalities by means of an analysis of the process and of a systematic review of the literature. The analysis is focused on countries with public organized screening programs.

## Methods

### Definition of the putative algorithm for HPV-based screening

After an initial search of the meta-literature (systematic reviews, HTA reports, guidelines, and narrative reviews) on implementing an HPV-based screening and social inequalities in cervical cancer prevention, we analyzed the putative screening algorithm with HPV as primary screening, as included in the up-coming European guidelines (9), to identify all the critical points where inequalities in coverage, participation, and compliance to protocols, and in the effectiveness of screening program could be generated or reduced.

### Scope

To define the scope of the systematic review we analyzed the flowchart of the algorithm to identify those points where new inequalities might be introduced and/or where existing inequalities might be reduced. Relevant topics should have two characteristics: (1) there should be a noticeable change with the introduction of the HPV-based screening; (2) the topic should be potentially related to socioeconomic inequalities. Among the parameters measuring the effectiveness of an organized screening program, test coverage, participation, and compliance have a “*per se*” effect on equity (9).

### Systematic review

#### Literature search

A systematic review of the literature was conducted on PubMed and the European public screening program websites to identify relevant publications on HPV screening and coverage, participation, and compliance.

The PubMed search strategy was defined according to the methods used in two previous systematic reviews, one on the methods to increase participation in screening (9, 22, 23) the other on inequalities and screening (24). To the original search of the systematic review on methods to increase participation we added terms to identify those focusing on inequalities as well as terms to specify HPV-based screening (HPV or human papillomavirus). The references of all the relevant papers were checked to identify other potentially relevant papers.

The search was limited to the period from 1/1/2000 to 31/7/2013. The rationale for limiting the search to 2000 is that in the 1990s there were no population-based experiences of HPV screening. Furthermore, the screening algorithms now proposed in organized screening were defined in the early 2000s (25).

#### Inclusion criteria

From the studies retrieved, we first selected those that were potentially relevant by reading the abstract. The abstract should present data or hypothesis on association or interaction between HPV knowledge, HPV screening attendance, and/or attitude and social, cultural, economic, and/or ethnic inequalities.

Due to resource limitations, the title and abstract screening was carried out by only one researcher (Flavia Baldacchini) and subsequently checked by a second author (Paolo Giorgi Rossi) by crosschecking the references. The full text of the relevant studies were then analyzed to identify and classify the information or hypothesis reported. A further number of studies was excluded in this step because the discussion of HPV and inequalities was too vague or because the paper focused exclusively on vaccination campaigns. The inclusion criteria were evaluated by two authors independently (Flavia Baldacchini and Paolo Giorgi Rossi); discordant cases were discussed to reach an agreement.

We did not perform any quality analysis since we were interested in identifying any issues that may be relevant, which could emerge even in low-quality papers.

#### Data extraction and narrative synthesis

From each paper the following data were extracted: publication year, author, title, topic(s) analyzed, outcome considered, study design, population, synthesis of results, and synthesis of the authors’ conclusions.

Information from the selected papers was extracted according to its relevance with the topics listed in the flowchart analysis. Topics that emerged from the literature search and not postulated in the flowchart analysis were also identified and reported in the results. The papers were also classified according to the outcome considered: knowledge, test coverage, screening attitude and/or screening participation, compliance to protocols, and anxiety. In addition, we considered papers concerning the interactions between vaccine and screening.

Systematic review results are summarized in a narrative synthesis.

## Results

### Flowchart analysis and search results

**Figure 1:**
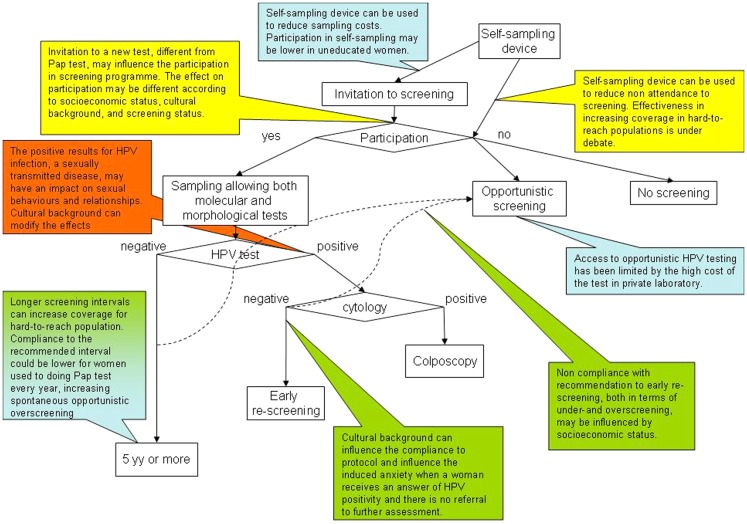
**Putative screening algorithm according to the up-coming European guidelines (9) and critical points related to social inequalities**. Along the pathways depicted by the screening algorithm flowchart, the callouts explain where critical points for increasing or decreasing social inequalities have been identified or hypothesized. The color code of the callouts corresponds to the main topics, as reported in Table 1 and Figure 2, to which the critical point refers: in blue, points related to test coverage of the target population; in yellow, points related to participation in screening programs; in green, points related to compliance with screening protocols; in orange, points related with communication of positive results and anxiety.

The critical points reported in Figure 1 were identified in the preliminary analysis of the meta-literature. They can be grouped into four main topics: test coverage of the population, participation in screening program, compliance to the screening protocols and referral strategies, and communication of positive results and related anxiety. Of the papers treating the effects on test coverage and screening participation, a relevant number focused on the possible use of self-sampling. Furthermore, two topics not directly linked to the screening protocol were identified: knowledge and communication strategies of HPV and the interaction between screening and HPV vaccine.

**Table 1:** **Summary of the questions and results emerged from the systematic review**.

Topic	Reference	Question	Summary of the results
Knowledge	(26–42)	Differences in knowledge	Almost all studies found a gradient in knowledge about HPV and cervical cancer with any of the variables used to measure SES or deprivation: educational level, disadvantaged groups, etc
	(43–48)	Effective tools to improve knowledge and reduce inequalities	Differences tend to diminish after any kind intervention to inform women. Results about written material are inconclusive; short counseling showed to be effective
Coverage	(16, 49–51)	Longer screening intervals can increase the coverage for hard-to-reach population	No data are available
	(9, 52)	Access to opportunistic HPV testing could be limited by the high cost of the test in private laboratories	No data are available
	(49, 53, 54)	Self-sampling device could be used to reduce sampling costs, but could affect coverage	Only one trial conducted in Mexico. A lower coverage was achieved, but it was due to women not found at home and to whom the self-sampling was not mailed
Participation	(9, 13, 16, 17, 21, 26, 32, 50, 52, 53, 55–57)	The proposal of a new test may change the participation	Several observational studies and one trial found a small increase in participation when invited for a HPV test compared to Pap test. No data on how it will impact on inequalities
		Longer intervals could disrupt the habits, thereby of women reducing participation	No data are available
	(14, 22, 49, 53–55, 58–75)	Self-sampling device could be used to participation in screening program	Nine studies showed a positive effect of self-sampling in under-screened population. Some studies showed that the device is acceptable also among disadvantage women, even in some case to a minor extent
Compliance to screening protocols	(41)	Longer intervals could increase opportunistic over-screening	No data are available
	(9, 21, 35, 36, 57)	Referral to early rescreen after HPV positive cytology-negative test could increase use of unnecessary ascertainment	No data are available
	(17, 21, 76, 77)	Referral to early rescreen after HPV positive cytology-negative test could result in substantial loss to follow up	The compliance to early rescreen in this group of women varied among studies, showing that correct communication can reduce loss to follow up. No data about differences in socio-economic status are available
Communication of positive results	(2, 9, 78–81)	Communication of HPV positivity can induce anxiety	Several studies showed anxiety after the communication of Pap test and/or HPV positivity. Women with higher educational level are advantaged in understanding the gynecologist’s and midwife’s recommendations and explanations
	(32, 40, 79, 80, 82–84)	Communication of HPV positivity can induce changes in sexual habits	Some studies showed concern about the sexual transmission of the virus. These concerns were stronger in women of lower educational level and disadvantaged ethnic groups
	(31, 36, 84–91)	Effective ways to reduce anxiety and other adverse effects	Face-to-face counseling was preferred by women, but cannot be used only for positive results because of the anxiety caused by the understanding that the test was positive and the need to wait for the counseling. Short phone counseling only for positive was also appreciated
Interaction vaccine and screening	(38, 92–98)	Negative effect of vaccine on future screening	Only one studies reported a small proportion of girls referring that vaccination would change their attitude to screening
	(56, 99–103)	Association between vaccine and screening attitudes	Several studies showed an association between vaccine and screening attitude or between vaccination and screening coverage in mothers. Results are heterogeneous

**Figure 2:**
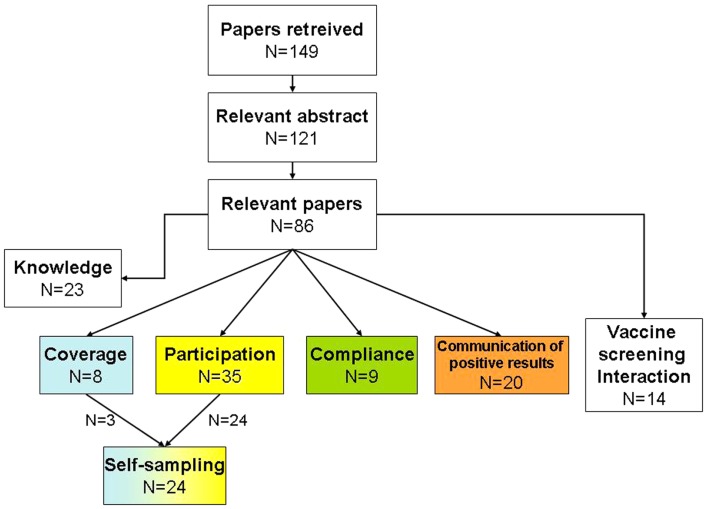
**Flowchart of the systematic search**. The color code of the main topics is the same as reported in Figure 1.

One hundred and forty-nine abstracts were initially identified. Of these, 121 were considered relevant, with 86 included after full text examination (Figure 2). During the literature analysis we summarized the information reported by each study according to the general issue for which it was relevant (Figure 1).

### Knowledge and communication strategies

The systematic review identified 23 studies on knowledge about HPV that reported differences in socio-economic status levels or interaction between SES and effectiveness of information/communication tools or strategies.

In general, the more deprived women, with lower educational level or SES, and women in more disadvantaged ethnic groups knew less about HPV and cervical cancer risk. Vanslyke and colleagues (26) found that knowledge of HPV was generally very limited among Hispanic women aged 18–60. Furthermore, an indirect evidence of inequality among Hispanics came from the comparison of the Health Interview National Trends Survey (HINTS) and a cross-section of callers to the National Cancer Institute’s (NCI) cancer information service (CIS) (27). Luque and colleagues (42) observed greater awareness of HPV and the HPV vaccine among Anglo American and Puerto Rican women than among Mexican and Honduran women. Al-Naggar and colleagues (28) examined the level of knowledge and barriers against cervical cancer screening of female university students in Selangor, Malaysia and found that age, marital status, ethnicity, and monthly family income, were significantly associated with knowledge of cervical cancer screening. Vogtmann and colleagues (29) evaluated the demographic and behavioral factors associated with HPV awareness and knowledge in a population of Mexican college students and found that characteristics associated with not having heard about HPV were being male, not having running water, not having health insurance, and not having sexual experience. In the UK, Waller and colleagues (30) found that women and more educated people had better knowledge of the established risk factors for cervical cancer and HPV and in Germany, Klug and colleagues (33) found an association with social class. Only one study on Turkish sexual workers found almost no association between HPV knowledge and educational level (34).

Most of the studies were conducted before the introduction of HPV vaccine. Indeed, it is very likely that the knowledge and awareness of HPV strongly increased in the years around the introduction of mass vaccination campaigns in 2007–2009. This was confirmed by some focus groups conducted with women participating in the Florence, Italy cervical cancer screening in 2007 and 2011 (35, 36). It is also likely that the increase in knowledge was more relevant for less educated women who started out with a lower level of knowledge. Nevertheless, the PREGIO study, conducted in Italy during the launch of the mass vaccination of 12-year-old girls in 2008, found that knowledge of Pap test was still greater than the knowledge of HPV and cervical cancer, although more than 70% correctly answered questions on virus transmission and the role of HPV in cancerogenicity (37). Women with higher educational level had greater knowledge about HPV and cervical cancer prevention but had similar attitudes toward undergoing vaccination. Two recent studies conducted in France (38) and Germany (39), instead, found there was still very little knowledge (16%) of the link between HPV and cervical cancer and an insufficient HPV awareness and low vaccination prevalence among young women.

Three trials and one case–control study compared different strategies to provide information on HPV. Lloyd and colleagues (43) observed an increase in knowledge in 13–16-year-old girls with an increase in fear but not in anxiety about infections after the distribution of a leaflet (not specific for HPV test and including other sexually transmitted diseases as well). Similar results were observed by Papa and colleagues (44) in women undergoing an HPV test and by Marek and colleagues (45) after a brief HPV-oriented program for adolescent. A limit of these two studies was the absence of information about SES. Wetzel and colleagues (46) evaluated the efficacy of a short counseling program and observed an increase in knowledge. The intervention was effective in reducing the existing differences between black and white women and between those with Medicaid and those with private insurance.

Other authors have tried to provide insights into effective communication about HPV, but with little supporting evidence (47, 48) and no mention of how to reduce SES inequalities.

### Coverage of the target population

Longer intervals will influence the test population coverage because the definition of test coverage will change, but also because there may be an impact on women’s behaviors and attitude. There are no direct evidences, experimental or observational, in the literature on this point.

Most of the literature is only speculative. Our systematic review retrieved eight studies on coverage and HPV-based screening that treated social inequalities. Some authors speculated on the effect of longer intervals, and the two options are reported (16, 49–51): longer intervals can facilitate achieving high-population coverage; longer intervals may disrupt women’s habits and thus have detrimental effects on coverage. The second hypothesis is usually supposed to affect especially deprived women (16).

Many countries have a mixed model with opportunistic and organized screening existing together and sometimes competing for participation (104, 105). Only two documents mention the consequences on social inequalities of the interaction between spontaneous and organized screening in the era of HPV-based screening (9, 52). The introduction of HPV as primary screening test may have some consequences in this context and can surely change the inequality scenario: in the opportunistic setting, HPV will be much more expensive than Pap test, at least in the short term. If opportunistic screening offers the two options, less wealthy women will be pushed to undergo Pap test and richer ones HPV, regardless of what would be more appropriate. If the diffusion of HPV testing in the opportunistic setting were stronger and the offer of Pap almost disappeared, the coverage in less wealthy women would decrease or these women would be induced to participate in organized screening programs. Finally, if the screening programs are reinforced by the introduction of HPV testing, it is likely that inequalities will decrease, as already observed in countries with well-established organized screening programs for cervical and breast cancer (106).

### Participation in screening program

The proposal of a new test may influence attendance. Thirty-five studies were found, but only one trial (53) and three intervention studies with historical controls (17, 21, 50) compared the participation in HPV-based vs. cytology-based screening program. The only trial (53) focused on self-sampling, but used as control groups both standard invitation to have a Pap test at the clinic and an invitation to have an HPV test at the clinic. The sample size was small and the population was selected to be non-responder to the first invitation, but it found an 8%, non-significant increase in participation. Data from pilot studies showed in most cases a higher participation in HPV-based screening than in Pap test-based screening. In particular, two Italian studies showed a 10% increase in coverage (17, 21). The increase was stronger in younger women, a group less covered than older women in Italy (17). Another study (50) found that after the initial diffidence, Hispanic and black women were more likely to undergo HPV test than were white women. Some other papers reported the results of pilot or demonstration studies but without any term of comparison. Levinson and colleagues (55) found high acceptability in underserved Peruvian women, Marlow and colleagues (56) found that, in contrast to screening attendance, ethnicity plays an important role in HPV testing, and finally, Philips and colleagues (13) report that adding HPV-based triage to the Pap program lowered the value of screening participation for only two women, whereas for the sample as a whole, it increased the average valuation by about 47%.

It is difficult to predict what impact the increase in participation will have on test coverage, but the increased costs in opportunistic screening and the increased appeal of programs offering the HPV test will probably increase participation in organized screening. Part of this increase will probably involve some women who are under- or never-screened, which will result in a substantial reduction in inequality of access and in the burden of disease (107).

### Use of self-sampling devices

Self-sampling devices can be used to increase coverage and/or participation among non-responders. Twenty-four studies treated the effect of self-sampling on coverage or participation. Nine trials (53, 54, 58, 60–65) and four reviews (22, 66–68) found a positive effect of direct home mailing of the device, while other ways of offering the device are not effective (53). Among studies reporting the acceptability of self-sampling among women, one showed that it was higher among married women and less accepted by some ethnic groups, such as Asians in the UK (69) and another found (73) that it was higher among those with some or more college education (43 vs. 26%), and among those who were not Hispanic compared to Hispanic (49 vs. 28%). However, four studies found high acceptability also in the most hard-to-reach women (67, 70, 71, 74). One of the main concerns in women performing self-sampling is not collecting an adequate sample (53, 69, 72, 75). This concern is stronger among Indian and African Caribbean women than among white British women (75). In particular, two trials in France conducted in two neighborhoods with different socio-economic levels (62, 64) measured the difference in effectiveness of self-sampling in increasing coverage: they observed that the relative risk of having a test was higher in low socio-economic status women but that the impact was limited in both contexts due to low participation and very low compliance to colposcopies in positive women. A study in the Netherlands (59), found that self-sampling was effective in increasing coverage among under-covered populations, but the response rate was higher in native-Dutch women and in women who already had a previous Pap test. Another study, conducted in central Italy, found no effect of self-sampling in a rural area and a relevant effect in urban areas (53), while a Swedish study found no difference in acceptance in different SES levels (108). These studies, designed to test self-sampling as a tool to improve Pap test-based programs, suggest that the most deprived women, even in HPV-based programs, will have more difficulty accepting self-sampling, but an increase in compliance can be obtained in most of the socio-economic groups.

Self-sampling devices can also be used to reduce sampling costs, though it could influence participation. Only one large trial in Mexico (49) tested self-sampling as first approach method and showed a lower participation for self-sampling than for Pap test when all the population was included in the analyses. If, however, we exclude women not found at home and to whom the self-sampling device was not mailed, the participation was over 98%. Such a result has the potential to eliminate any inequality in access to screening, providing we have complete lists of resident women with updated and accurate addresses.

### Compliance to screening protocols

Nine papers treated the differences in compliance with respect to HPV-related protocols.

Compliance to a 5-year interval after HPV-negative test may be low for women used to having a Pap test every year (41), thereby increasing spontaneous opportunistic over-screening. No published data were found about compliance to the recommended interval after HPV. Previous studies found an association between intervals shorter than recommended and high socio-economic status women (109, 110). Consequently, if over-screening increases, the phenomenon will probably be less relevant for deprived women.

Non-compliance with the recommendation to repeat test after 1 year (or 6 months), both in terms of under- and over-screening, may be influenced by socio-economic status. It has been observed that women have an overwhelming preference for immediate colposcopy rather than continued surveillance for persistent HPV (57). Cultural background can influence the compliance to protocol and increase or decrease the induced anxiety when a woman receives a result of HPV positivity and there is no immediate referral to further assessment.

Some authors considered the implication of effective communication of the test results on compliance to 1-year follow up (9, 21). The implications differ depending on whether it is necessary to invite the women back to collect a cytologic sample or whether the sampling method allows reflex cytology in to be performed (a liquid-based cytology or a double sampling taken at the same moment). In the first case, high compliance is needed to obtain the second sample. If the Pap test is negative, women who are HPV positive and Pap test negative must be reassured about waiting 1 year before repeating the test so as to avoid unnecessary colposcopies or other evaluations during that period. In the case of a reflex cytology test, we can give the HPV and cytology results at the same time, leaving only the problem of reassuring women for the 1-year follow up. Compliance to protocols varied dramatically among trials and pilot studies results, with some programs obtaining more than 85% without any particular interventions (17) and other pilot studies (21) and trials with relevant loss to follow up in HPV positive women (76, 77) when colposcopy was delayed. In some cases there was an evidence of over-screening (21), but there are no data on SES differences. In a non-randomized study, a brief phone counseling at the moment of HPV positivity strongly increased the compliance (65 vs. 35%) to 1-year follow up (21).

All the problems related to compliance to recommended protocols are more critical when a strong opportunistic offer is present (52, 111), in particular when there is a risk of overuse of screening test (i.e., shorter intervals or extra Pap test in HPV-negative women) and of evaluations (i.e., extra colposcopies in HPV positive cytology-negative women). In fact, in opportunistic screening gynecologists usually recommend a yearly Pap test and women are used to shorter intervals than that recommended by organized screening. In some focus groups (35, 36), a certain diffidence against longer intervals emerged when proposed by the public health system because it was perceived as a budget cut rather than a measure to avoid overtreatment (112). Even if this sort of skepticism may be present in all cultural and economic strata of society, the effect on compliance will probably be greater among women with higher SES because they undergo regular gynecological exams more frequently than do women with low SES.

The only way to reduce negative interactions between organized and opportunistic screening during the HPV screening implementation is to reduce discrepancies between organized program protocols and the attitudes/recommendations of gynecologists and general practitioners working outside the program, be they public or private providers. How to achieve this is beyond of the scope of this paper, but education and participation in the definition of local protocols, within the limits imposed by the evidence, are necessary steps in the process (113, 114).

### Communication of positive results and related anxiety

Finally, the communication of HPV infection positivity poses new problems combining the anxiety and the implications related to a sexually transmitted disease with that related to cancer (79, 80). We found 20 papers on the communication of positive results and how a woman’s socio-economic status can affect how she receives it.

Previous systematic review (2, 9, 78) found that communicating the result of an abnormal Pap smear may induce anxiety, fear of malignancy, difficulties in sexual intercourse, a different perception of the body, and fear of becoming infertile (115–118). Furthermore, some women reported fear of pain caused by colposcopy and treatment, which may cause loss to follow up (117). One paper shows that the anxiety induced by positive Pap test results (81) was stronger in women with low socio-economic status. Indeed, we can suppose that many of the feelings related to a positive result are determined by the woman’s knowledge and ability to understand the midwife’s or gynecologist’s indications, which is almost certainly associated with her educational level. All these concerns are also valid in HPV-based screening and may be even more intense given the higher proportion of positive women (82). Furthermore, there are concerns that are unique to HPV-based screening: since this test explicitly targets a sexually transmitted virus, positivity may have an impact on sexual behaviors and relationships (40, 82). This concern has been shown to be higher in low education level women and non-white ethnic groups in the UK (32), and more in general, to women’s social status (married vs. unmarried), sexual history (number of partners), cultural background (sexual norms and practices), and knowledge and understanding of the link between HPV and cancer (83). Several studies dealing with this found that current and past relationships, cultural norms concerning sexual habits, and knowledge of HPV and cervical cancer were possible modifiers of the psychological response to a positive result (82). Two studies using focus groups to evaluate the health education material and response letters of a pilot screening program found that the women asked for shorter texts and simpler wording; the greatest concerns were related to the difficulty in understanding the real risk of cancer (36, 87). Finally, Waller et al. (31) concluded that the way in which information is presented to women may be crucial in minimizing the negative psychological impact of testing positive and ensuring that participation in screening remains high.

Eight studies analyzed the health information needs of women undergoing an HPV test and in particular, of those receiving a result of positivity (84–91). Only one, based on Pap test experience, reports the results of different communication strategies. However, given the low number of participants (20 women), the authors could not determine whether effectiveness was associated with SES. Women questioned whether communicating results by letter would violate their privacy and generally preferred receiving a phone call because it permits immediate clarification, thereby reducing anxiety. The most preferred mode was face-to-face communication, for negative and positive results alike. In fact, they were worried that a differentiated mode of communication (letter for negative results and face-to-face for positive results) would increase their anxiety because the amount of time that elapsed from the date of the appointment would implicitly mean a positive result, to be confirmed or not during the counseling itself (84).

Obviously, a phone call and face-to-face communication make it possible to modulate the message according to the woman’s coping, linguistic abilities, and educational level. However, there are two main obstacles to implementing face-to-face communication: it is very time-consuming and, to avoid increasing anxiety, it should be done for both negative and positive results.

### Vaccine and screening interaction

One of the most treated topics in the literature was the interaction between vaccination and screening, with 14 papers directly reporting data on this topic. Two main points were treated: (1) the effect of vaccination on women’s attitude to screening and consequently on test coverage (92–94, 96–98), the hypothesis is that being vaccinated could change future participation in screening [usually the authors suggest a decrease – Ref. (93, 94)]; (2) the association between vaccination and future screening attitude (56, 99–102), i.e., those girls who do not do the vaccination now are probably those who will not participate in screening in the future. The consequence is that the impact on cervical cancer screening of vaccinated cohorts will be minimal because most cancers will occur in women not vaccinated and not screened (95). These two phenomena are not typical of the HPV-based screening and most of the literature considered the relation between vaccination and Pap test use.

The first point has been treated by Brotherton and colleagues, with interviews to girls to whom vaccination had been proposed: only 8% said that HPV vaccine could have a negative effect on their future screening habits.

As for the second point, two study designs have been used: attitude interviews to vaccinated and unvaccinated girls already in or close to the screening target age (99, 103); for younger girls, the association between the mother’s Pap test use and the daughter’s vaccination state (56, 100, 101). The results are mixed: two studies (56, 99) found no association while the others found a strong association between vaccination and screening (99) or between mother’s participation in screening and her daughter’s being vaccinated (100–102).

None of the studies hypothesized how the introduction of HPV test might modify these phenomena. Only the study by Marlow and colleagues explicitly measured attitudes to HPV testing.

The general opinion is that HPV should be the primary screening test in vaccinated cohorts (119) and research on vaccine and screening interaction should be directed to define the best screening algorithms for vaccinated women.

## Conclusion

Since equity in access is one of the main objectives of organized screening programs, the introduction of HPV test may be a way to increase population coverage and thus finally include some of the hard-to-reach women. Interactions with spontaneous screening may increase over-screening and inappropriate use of evaluation tests although this phenomenon is likely to be more relevant among women with higher socio-economic status.

There remain, however, problems concerning how to communicate positive results, control induced anxiety, and reduce non-compliance to protocols, which may augment existing inequalities if particular attention is not paid to effective communication.

## Conflict of Interest Statement

Paolo Giorgi Rossi is the principal investigator of a project sponsored by the Italian Ministry of Health and data owner. For this project, Paolo Giorgi Rossi is in contact with Roche Diagnostics, Hologic Gen-Probe, Qiagen, Abbott, for tests at reduced price or free of cost. All other authors have no conflicts of interest.

## Supplementary Material

The Supplementary Material for this article can be found online at http://www.frontiersin.org/Journal/10.3389/fonc.2014.00020/abstract
